# Supplementary material for: A POLD3/BLM dependent pathway handles DSBs in transcribed chromatin upon excessive RNA:DNA hybrid accumulation
Source: Nat Commun. 2022 Apr 19;13:2012. doi: 10.1038/s41467-022-29629-2 (PMC9019021; doi:10.1038/s41467-022-29629-2)
Supplement: Supplementary file 1 — Supplementary Information [file 41467_2022_29629_MOESM1_ESM.pdf]

Supplementary information

A POLD3/BLM dependent pathway handles DSBs in transcribed chromatin upon excessive RNA:DNA hybrids accumulation.

Cohen S[1#], Guenolé A[1#], Lazar I[1#], Marnef A[1], Clouaire T[1], Vernekar DV[2], Puget N[1], Rocher V[1], Arnould C[1] Aguirrebengoa M[1], Genais M[1], Firmin N[1], Shamanna RA[3], Mourad R[1], Bohr VA[3], Borde V[2] and Legube G[1]*.

1. MCD, Centre de Biologie Integrative (CBI), CNRS, Université de Toulouse, UT3

2 Institut Curie, Université PSL, Sorbonne Université, CNRS UMR3244, Dynamics of Genetic Information, Paris, France

3. Section on DNA Repair, National Institute on Aging, National Institutes of Health, Baltimore, MD, USA

Supplementary Figures 1-7

Supplementary Table 1-2

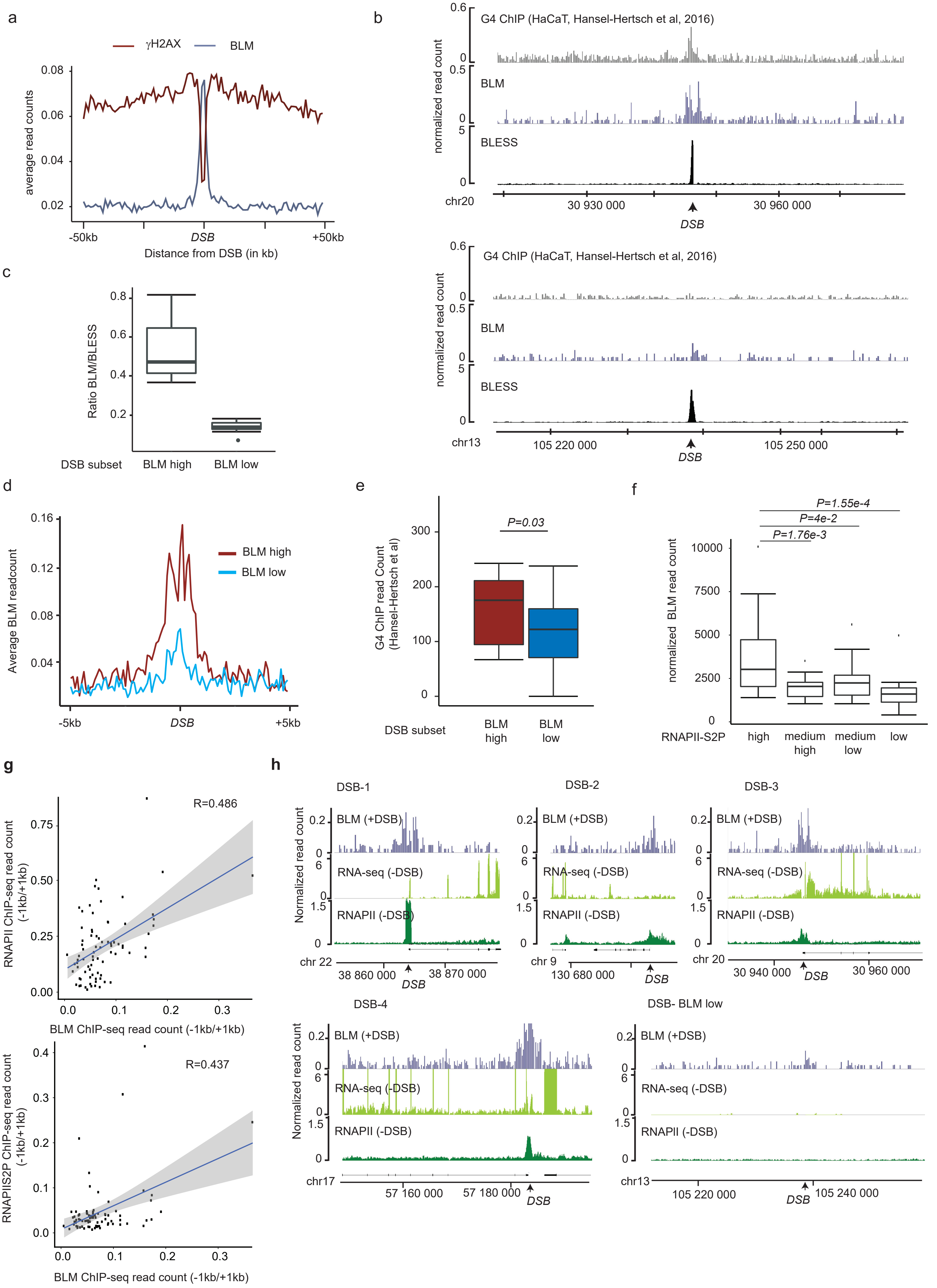
a
γH2AX
BLM
average read counts
0.08
0.06
0.04
0.02
-50kb
DSB
+50kb
Distance from DSB (in kb)
b
normalized read count
G4 ChIP (HaCaT, Hansel-Hertsch et al, 2016)
BLM
BLESS
chr20
30 930 000
30 960 000
DSB
G4 ChIP (HaCaT, Hansel-Hertsch et al, 2016)
BLM
BLESS
chr13
105 220 000
105 250 000
DSB
c
Ratio BLM/BLESS
DSB subset
BLM high
BLM low
d
Average BLM readcount
BLM high
BLM low
-5kb
DSB
+5kb
e
G4 ChIP read Count (Hansel-Hertsch et al)
P=0.03
DSB subset
BLM high
BLM low
f
normalized BLM read count
P=1.55e-4
P=4e-2
P=1.76e-3
RNAPII-S2P
high
medium high
medium low
low
g
RNAPII ChIP-seq read count (-1kb/+1kb)
R=0.486
BLM ChIP-seq read count (-1kb/+1kb)
RNAPIIS2P ChIP-seq read count (-1kb/+1kb)
R=0.437
BLM ChIP-seq read count (-1kb/+1kb)
h
DSB-1
DSB-2
DSB-3
Normalized read count
BLM (+DSB)
RNA-seq (-DSB)
RNAPII (-DSB)
chr 22
38 860 000
38 870 000
DSB
chr 9
130 680 000
DSB
chr 20
30 940 000
30 960 000
DSB
DSB-4
DSB- BLM low
chr17
57 160 000
57 180 000
DSB
chr13
105 220 000
105 240 000
DSB

Supplementary Figure 1. BLM ChIP-seq profile at DSBs.

a. Average profiles of γH2AX (red) and BLM (blue) ChIP-seq obtained after DSB induction (4h) for the 80 best-induced DSBs (100 kb window). **b.** Genomics tracks of BG4 ChIP-seq (grey, from[56]), BLM ChIP-seq (blue), BLESS (black) at two DSBs cleaved at an equivalent level (chr20: 30946312, enriched in BLM and chr13:105238551, not enriched in BLM). **c.** Boxplot showing the ratio BLM/BLESS signal on BLM-high (left, n=20) and BLM-low (right, n=20) DSB sites in treated DIvA cells. Center line: median; Box limits: 1st and 3rd quartiles; Whiskers: Maximum and minimum values, Points: outliers. **d.** Average profiles of BLM on BLM-high (red, n=20) and BLM-low (blue, n=20) DSBs (10kb window). **e.** Boxplot representing G4 ChIP-seq read count[56] (SRE5586987) on a 1kb window for BLM-high (red, n=20) and low (blue, n=20) DSB sites. Center line: median; Box limits: 1st and 3rd quartiles; Whiskers: Maximum and minimum values. P=0.035 (non parametric Wilcoxon test, two-sided). **f.** Boxplot representing normalized BLM ChIP-seq read count on 5kb around DSB after DSB induction on loci with high (n=20), medium high (n=20), medium low (n=20), or low (n=20) RNAPII-S2P occupancy before DSB induction (determined by ChIP-seq before DSB induction). Center line: median; Box limits: 1st and 3rd quartiles; Whiskers: Maximum and minimum values, Points: outliers. *P,* non parametric Wilcoxon test, two-sided. **g.** Scatterplot showing the correlation between BLM ChIP-seq and RNAPII (top panel) or RNAPIIS2P (bottom panel) on 2kb around DSB. R (Pearson) is indicated. Error in grey represents the regression confidence as obtained by the geom_smooth function of ggplot2 (linear model). **h**. Genome Browser screenshot of the BLM ChIP-seq signal (purple) in DIvA cells (4h post DSB induction), together with the RNA-seq (light green) and total RNAPII ChIP-seq (green) signals obtained in DIvA cells prior DSB induction[18] at five DSBs. DSBs 1 to 4 are further used in this study.

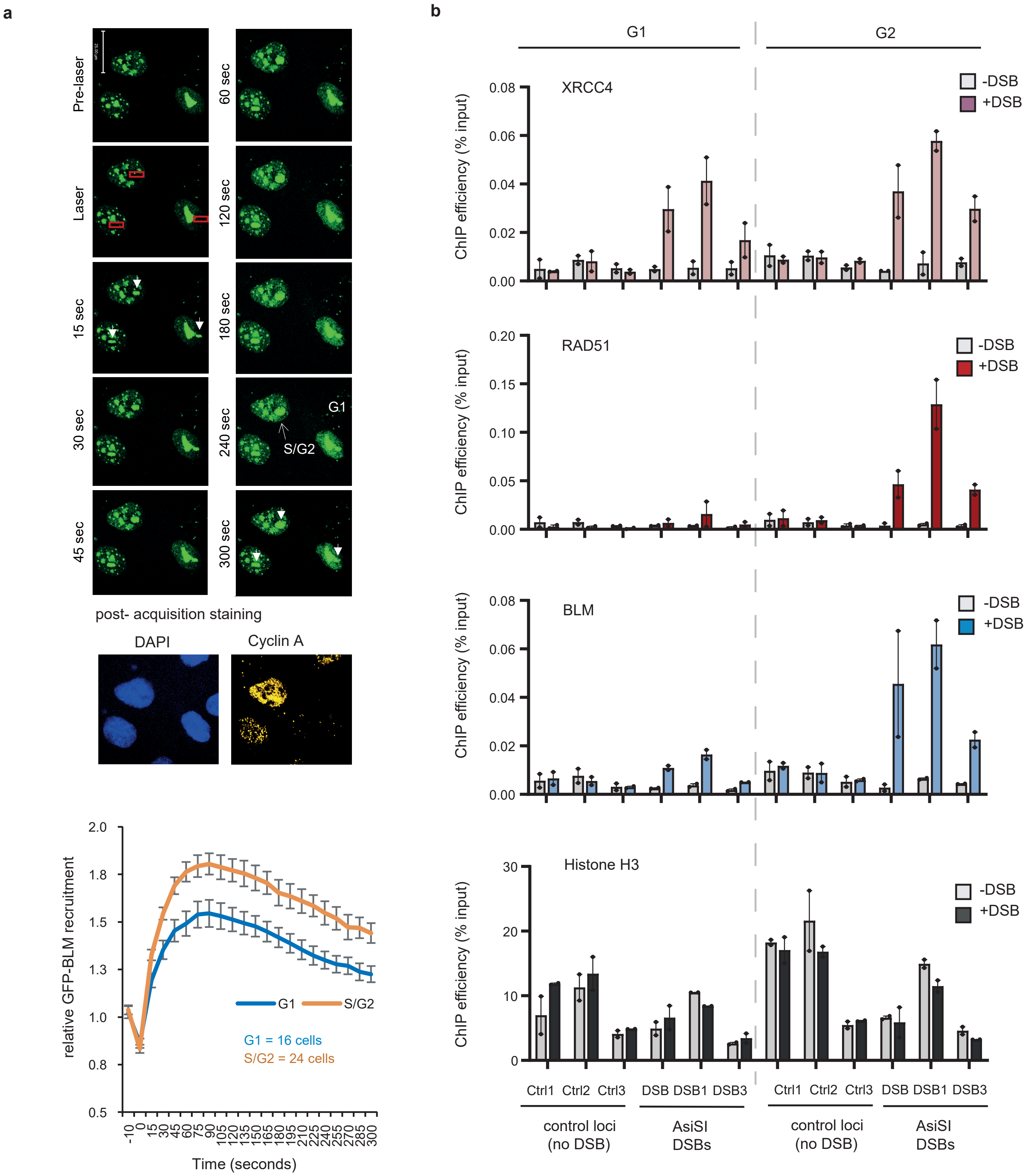

Supplementary Figure 2: cell cycle dependency of BLM recruitment

a. Kinetics of GFP-tagged BLM recruitment at sites of microirradiation in G1 and G2 cells, as determined by cyclin A2 immunostaining. Scale bar, 25µM. Bottom panel shows quantification as mean and SEM of n=16 (G1) and n=24 (G2) cells. A representative experiment is shown **b.** ChIP against XRCC4, RAD51, BLM and Histone H3 as indicated in DIvA cells before and after DSB induction (4h) following synchronization (double thymidine block). Enrichment (% of input) was quantified by qPCR at 3 control locus (no DSB) and 3 AsiSI-DSBs. (DSB chr1: 89458597; DSB-1 chr22:38864101; DSB-3 chr20: 30946312). Data are presented as mean and S.E.M of n=2 biologically independent experiments.

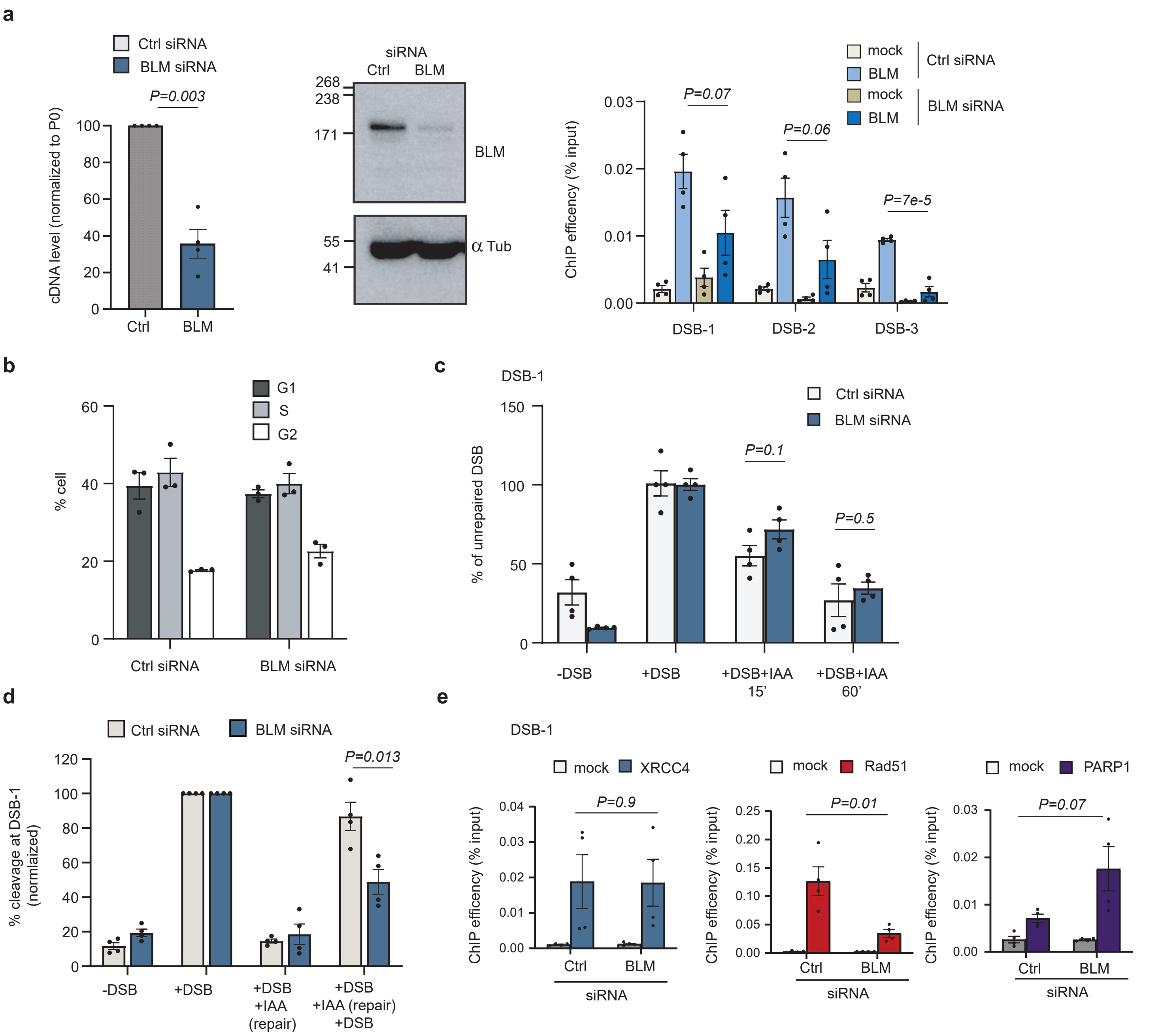

Supplementary Figure 3. BLM fosters resection, RAD51 loading and repair fidelity

a. Left panel, normalized cDNA level in control and BLM siRNA transfected DIvA cells. Mean and SEM of n=4 biologically independent experiments are plotted. *P*, paired t-test (two-sided). Middle panel, immunoblot (using Tubulin and BLM antibodies) following BLM and control siRNA transfection as indicated. Right panel, BLM and mock (no antibody) ChIP in DIvA cells after DSB induction (4h) transfected with control and BLM siRNA as indicated. BLM enrichment was assessed by qPCR at three DSBs showing BLM signal by ChIP-seq (DSB-1 chr22:38864101; DSB-2 chr9:130693170, DSB-3 chr20: 30946312). Mean and SEM of n=4 technical replicates are plotted. *P*, unpaired t-test (two-sided). **b.** Cell cycle distribution of control and BLM siRNA-depleted DIvA cells as indicated. Mean and SEM are indicated for n=3 biologically independent experiments. **c.** Repair kinetics measured by qPCR (see methods) at DSB-1 (chr22:38864101) in control or BLM siRNA-transfected cells, at different times points after auxin addition as indicated. Mean and SEM of n=4 technical replicates of a representative experiment are shown. *P*, unpaired t-test (two-sided). **d.** Cleavage efficiency at DSB-1 (chr22:38864101), showing the percentage of cleavage (normalized) in control (grey) and BLM (blue) siRNA depleted AID DIvA cells before DSB (-DSB), after DSB (+DSB), after repair (+DSB+IAA(repair)) and after a second round of DSB induction (+DSB+IAA(repair)+DSB). Mean and SEM of n=4 technical replicates of a representative experiment are shown. *P*, unpaired t-test (two-sided). **e.** RAD51, PARP1 and XRCC4 recruitment at DSB was assessed by ChIP in DIvA cells after DSB induction (4h) in control or BLM siRNA transfected cells. qPCR data are shown as percent of input immunoprecipitated at 80bp (XRCC4) and 800bp (PARP1, RAD51) from DSB-1 (chr22:38864101). Mean and SEM of n=4 technical replicates of a representative experiment are shown. *P*, unpaired t-test (two-sided).

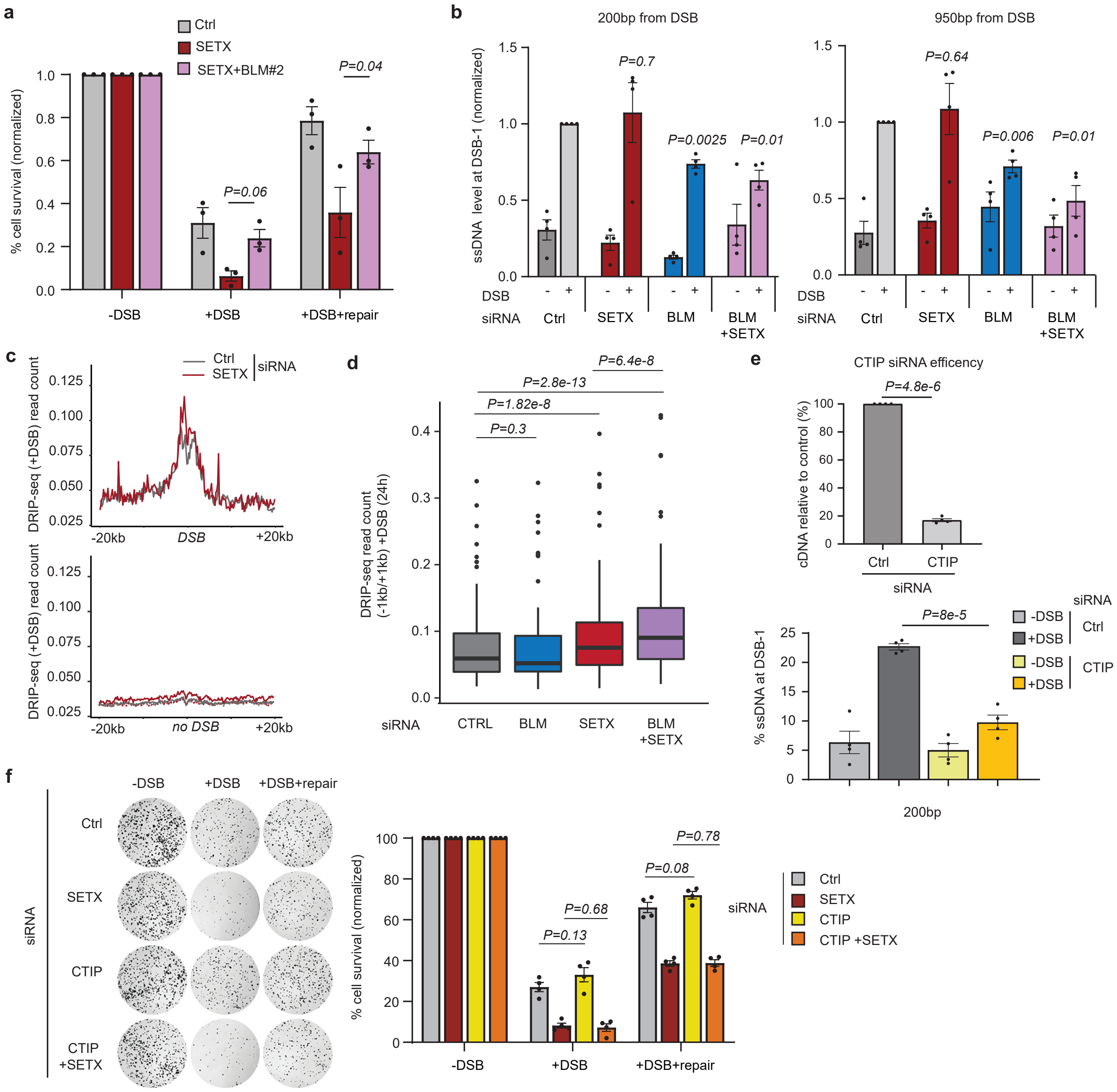

Supplementary Figure 4. Resection, RNA:DNA hybrids levels and cell survival in BLM/SETX deficient cells

a. Clonogenic assay in control, SETX, and BLM#2/SETX siRNA depleted AID-DIvA cells before (-DSB), after (+DSB) DSB induction and after DSB induction and auxin treatment (+DSB+repair) as indicated (left panel). Quantification (right panel) shows the percentage of cell survival (mean and SEM for n=3 biologically independent experiments). *P*, paired t-test (two-sided). **b.** Resection assay showing the level of single strand DNA before and after DSB induction for 4h (+DSB) in control, SETX, BLM, and BLM/SETX siRNA depleted DIvA cells at 200 bp (left panel) and 950 bp (right panel) from a DSB bound by BLM (DSB-1 chr22:38864101). Mean and SEM of normalized values (to control +DSB) of n=4 biologically independent experiments are plotted. *P*-values, one sample t-test, assessing significance with siRNA Ctrl +DSB (μ=1). **c.** Average DRIP-seq profiles post DSB induction (24h) around the 80 best cleaved DSBs (top panel) and 80 random sites (no DSB, bottom panel) in DIvA cells transfected with control and SETX siRNA. **d.** Box plot showing DRIP-seq read count after DSB induction, on a -1kb/+1kb window around the 80 best DSBs, in DIvA cells transfected with Ctrl, BLM, SETX or BLM+-SETX siRNA as indicated. *P*, non parametric Wilcoxon test (two-sided). Center line: median; Box limits: 1st and 3rd quartiles; Whiskers: Maximum and minimum values, Points: outliers, n=80. **e.** Upper panel: Normalized cDNA level showing CTIP siRNA-mediated knockdown efficiency. Mean and SEM for n=4 technical replicates. *P* unpaired t.test (two-sided). Lower panel: Resection assay showing the level of single-strand DNA (normalized to control +DSB) in control and CTIP-depleted DIvA cells at 200 bp from DSB-1 (chr22:38864101). Mean and SEM for n=4 technical replicates of a representative experiment is shown. *P,* unpaired t-test (two-sided). **f.** Clonogenic assay in control, SETX, CTIP, and CTIP/SETX siRNA depleted AID-DIvA cells before (-DSB), after (+DSB) DSB induction and after DSB induction and auxin (IAA) treatment (+DSB+repair) as indicated (left panel). Quantification (right panel) shows the percentage of cell survival (mean and SEM of n=4 biologically independent experiments). *P*, paired t-test (two-sided).

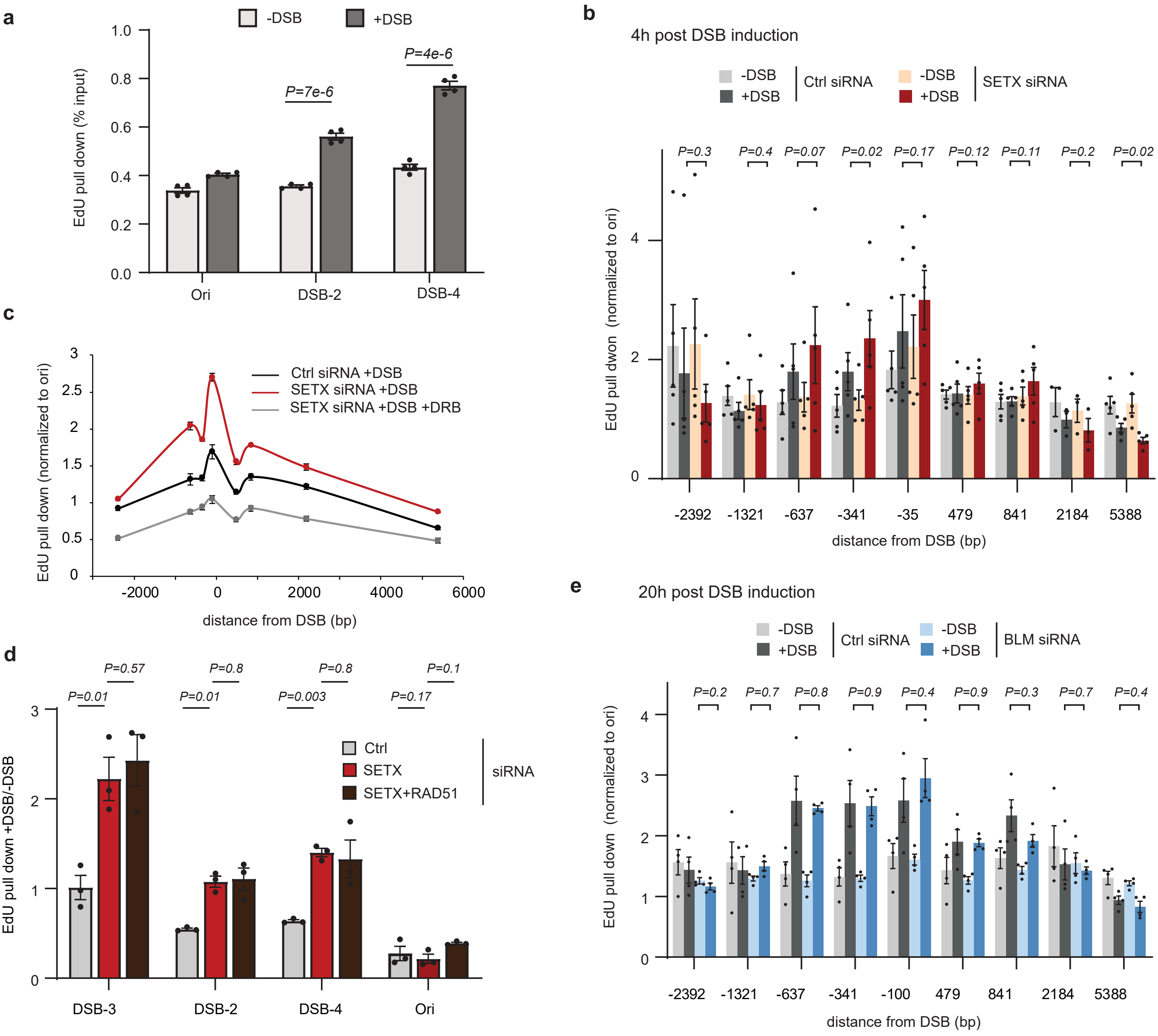

Supplementary Figure 5. Repair associated DNA synthesis at DSBs

a. EdU-pull down efficiency before (-DSB) and after (+DSB) DSB induction for 4h at an origin of replication (Ori; used as a control region to measure the background of DNA synthesis due to DNA replication) and at two DSBs bound by BLM (DSB-2, chr9:130693170, DSB-4, chr17: 57184296). Data are shown as percent of input pull-down at the three genomic locations. Mean and SEM of n=4 technical replicates. *P*, unpaired t-test (two-sided). **b.** Repair synthesis measured by EdU-pull down on a -/+ 3kb region around the DBS-2 (chr9:130693170) in control and SETX siRNA-depleted, before and after DSB induction as indicated (4h DSB induction). Data are shown as values normalized to the Ori region, serving as a control of EdU pull down efficiency (see Supplementary Fig. 5a). Mean and SEM of b=5 biologically independent experiments are shown. *P*, paired t-test (two-sided), between Ctrl and SETX siRNA +DSB. **c.** Repair synthesis profile measured by EdU-pull down after DSB induction (4h) on a -/+ 3kb region around the DBS-2 (chr9:130693170) in control and SETX siRNA-depleted, without treatment or with a treatment with DRB, as indicated. Data are shown as values normalized to the Ori region, serving as a control of EdU pull-down efficiency. Mean and SEM of n=3 technical replicates of a representative experiment is shown. **d.** Repair synthesis measured by EdU-pull down at 3 TC-DSBs and the Ori control genomic locus, in Ctrl, SETX and SETX+RAD51 siRNA-depleted cells. Mean and SEM of n=3 biologically independent experiments are shown. *P,* paired t-test (two-sided). **e.** Repair synthesis measured by EdU-pull down on a -/+ 3kb region around the DBS-2 (chr9:130693170) in control and BLM siRNA-depleted, before and after DSB induction (20h) as indicated. Mean and SEM of n=4 biological replicates are shown. *P*, paired t-test (two-sided), between Ctrl and BLM siRNA +DSB.

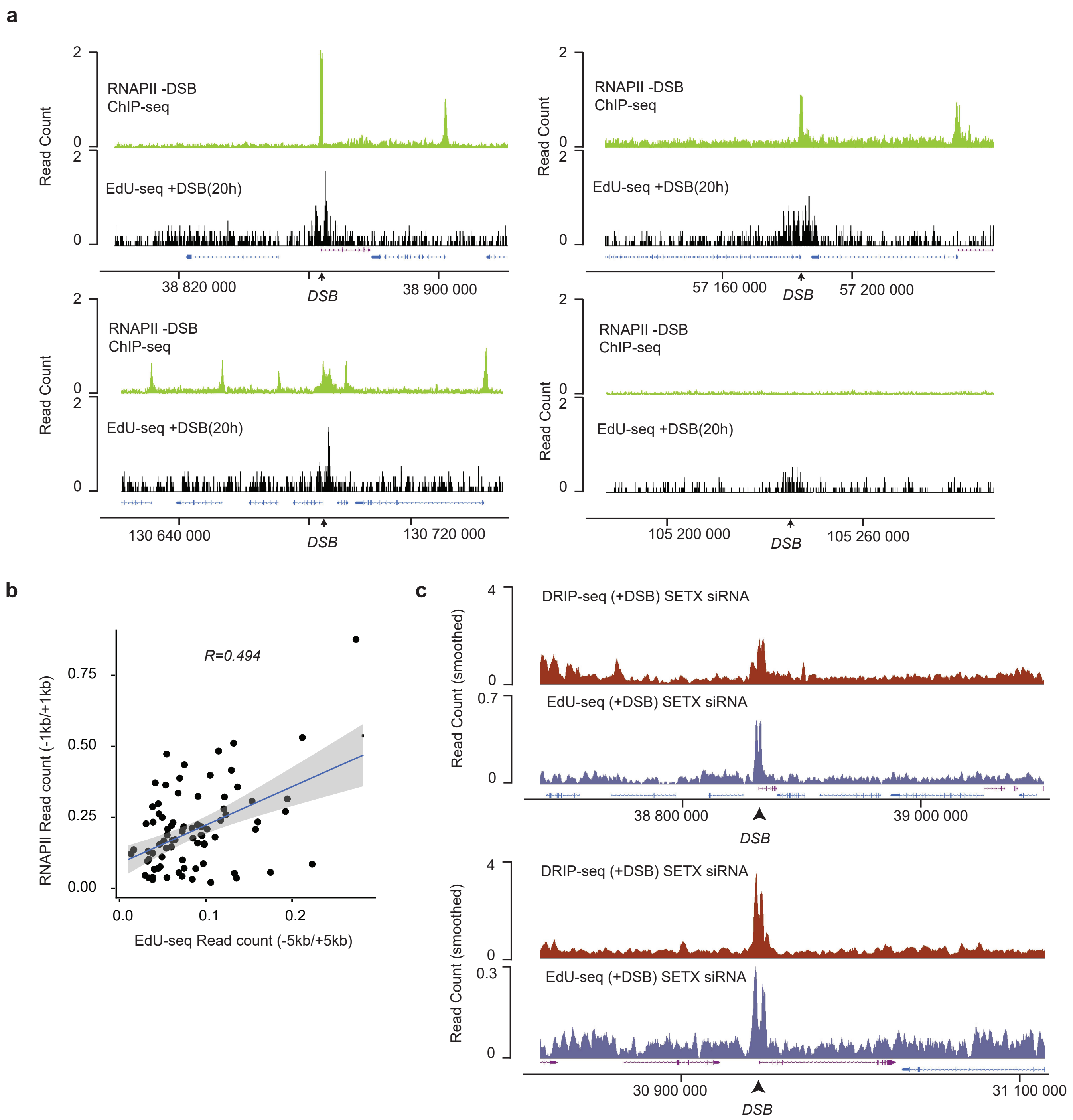

Supplementary Figure 6. Repair associated DNA synthesis by EdU-seq

a. EdU-seq genomic tracks (black) obtained after 20h of DSB induction in mimosin-treated cells. RNAPII ChIP-seq track is also shown on top (green). Three DSBs induced in active loci (top three panels) and one in an inactive locus (bottom panel) are shown. DSB are indicated by arrows. **b.** Scatterplot showing the correlation between EdU-seq read count (-5/+5kb) and RNAPII read count (-1/+1kb) at each DSBs induced by AsiSI in the DIvA system (80 best DSBs). R, pearson. Error in grey represents the regression confidence as obtained by the geom_smooth function of ggplot2 (linear model). **c.** Genomic tracks showing EdU-seq and DRIP-seq read count (smoothed on 1kb) obtained in SETX- depleted cells, at two TC-DSBs.

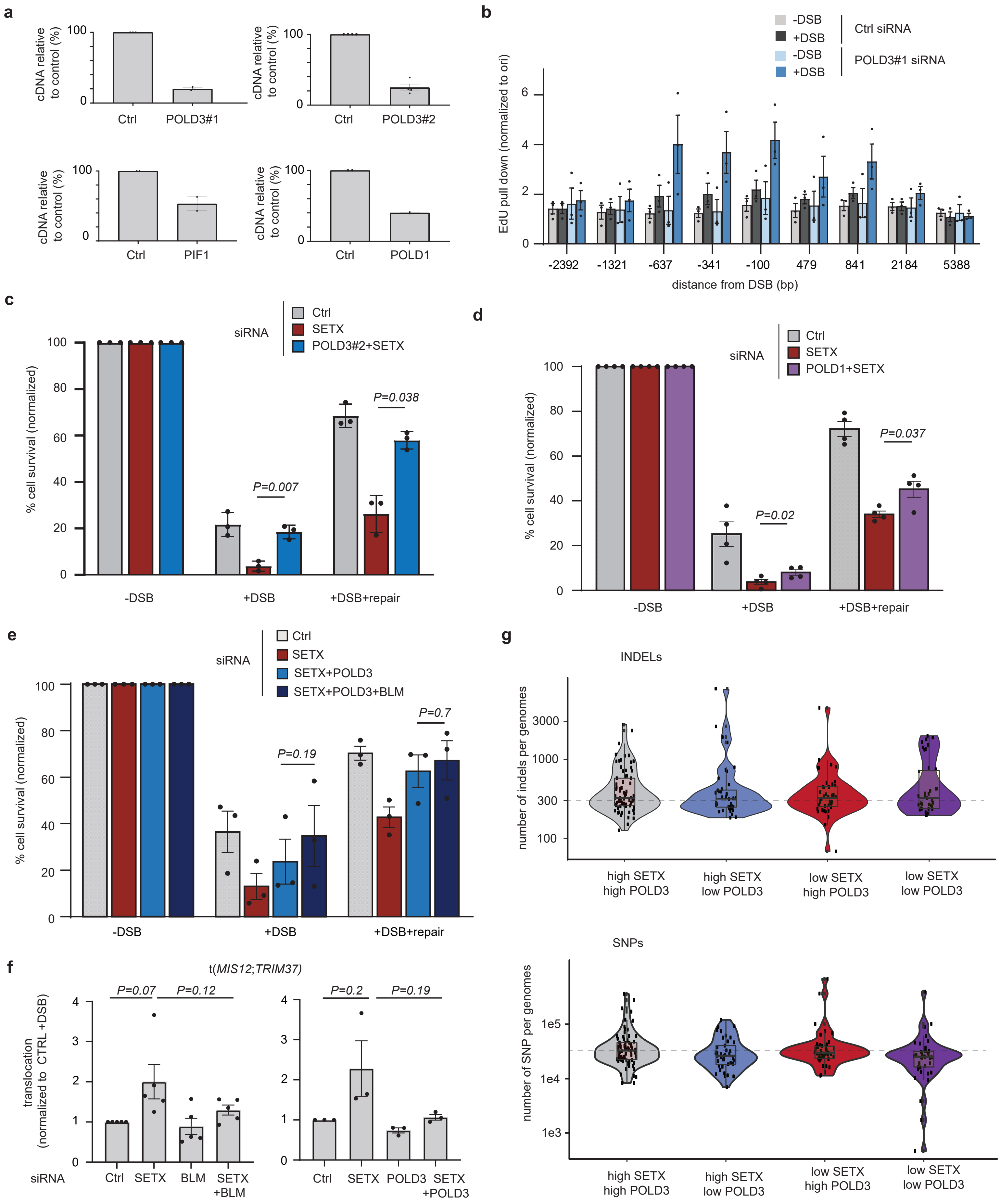
a
cDNA relative to control (%)
100
0
Ctrl
POLD3#1
POLD3#2
PIF1
POLD1
b
-DSB
+DSB
Ctrl siRNA
POLD3#1 siRNA
EdU pull down (normalized to ori)
-2392
-1321
-637
-341
-100
479
841
2184
5388
distance from DSB (bp)
c
siRNA
Ctrl
SETX
POLD3#2+SETX
% cell survival (normalized)
P=0.007
P=0.038
-DSB
+DSB
+DSB+repair
d
POLD1+SETX
P=0.02
P=0.037
e
SETX+POLD3
SETX+POLD3+BLM
P=0.19
P=0.7
f
t(MIS12;TRIM37)
translocation (normalized to CTRL +DSB)
P=0.07
P=0.12
P=0.2
P=0.19
BLM
SETX +BLM
POLD3
SETX +POLD3
g
INDELs
number of indels per genomes
high SETX high POLD3
high SETX low POLD3
low SETX high POLD3
low SETX low POLD3
SNPs
number of SNP per genomes
1e5
1e4
1e3

Supplementary Figure 7: POLD3 in DNA synthesis at TC-DSBs.

a. Normalized cDNA level showing POLD3, PIF1, and POLD1 siRNA-mediated knockdown efficiency as indicated. Mean and SEM of biologically independent experiments are plotted. POLD3#1, n=3; POLD3#2, n=3; PIF1, n=2; POLD1, n=2. **b.** Repair synthesis measured by EdU-pull down on a -/+ 3kb region around the DBS-2 (chr9:130693170) in control and POLD3 siRNA-depleted, before and after DSB induction (20h) as indicated. Mean and SEM of n=3 biologically independent experiments are shown. *P*, paired t-test (two-sided). **c.** Percentage of cell survival from clonogenic assay in control, SETX and POLD3#2/SETX siRNA depleted AID-DIvA cells before (-DSB), after (+DSB) DSB induction (4h) and after repair (+DSB+repair). Mean and SEM are plotted, n=3 biologically independent experiments. *P*, paired t-test (two-sided). **d.** Percentage of cell survival from clonogenic assay in control (Ctrl), SETX and POLD1/SETX siRNA depleted AID-DIvA cells before (-DSB), after (+DSB) DSB induction and after repair (+DSB+repair). Mean and SEM are plotted, n=4 biologically independent experiments. *P,* paired t-test (two-sided). **e.** Percentage of cell survival from clonogenic assay in control (Ctrl), SETX, POLD3/SETX and POLD3/BLM/SETX siRNA depleted AID-DIvA cells before (-DSB), after (+DSB) DSB induction and after repair (+DSB+repair). Mean and SEM are plotted, n=3 biologically independent experiments. *P*, paired t-test (two-sided). **f.** Translocation frequency t (*MIS12;TRIM37*) analyzed by qPCR in AID-DIvA cells transfected with siRNA as indicated, after DSB induction and repair. Mean +/- SEM are plotted. Left panel, n=5 biologically independent experiments; right panel, n=3 biologically independent experiments. *P*, paired t-test (two-sided). **g.** Top panel: Violin Plots representing the number of SNPs per genome depending on SETX and POLD3 expression as indicated using pancreatic cancer gene expression and genomic data available on ICGC database (PACA-CA project). From left to right, n=76; n=41; n=41; n=40. Center line: median; Box limits: 1st and 3rd quartile; Whiskers: Maximum and minimum values without outliers. Bottom panel: Violin Plots representing the number of INDELs per genome depending on SETX and POLD3 expression as indicated using pancreatic cancer gene expression and genomic data available on ICGC database (PACA-CA project). From left to right, n=75; n=40; n=40; n=39. Center line: median; Box limits: 1st and 3rd quartile; Whiskers: Maximum and minimum values without outliers.

BLM	AGCAGCGAUGUGAUUUGCAtt.
BLM #2	GCAUCUGACCAUCUGUGACUAtt
SETX	GAGAGAAUUAUUGCGUACUtt
CtIP	GCUAAAACAGGAACGAAUCtt
POLD3	Silencer® Select validated siRNA Ambion s21045
POLD3 #2	GAUCAGAGACUUGGAGUGGUCAAtt
PIF1	CCCUUCAGAGCCUAACCAAtt
POLD1	GGGACCAGGGAGAAUUAAUtt
RAD51	CCAGAUCUGUCAUACGCUAtt

Suplementary Table 1: siRNA sequences used in this study

	Location of the genomic feature (hg19)	FW	REV	distance to DSB	application	related Figure(s)
**DSB-1**	chr22:38864102 38864108	CCGCCAGAAAGTTTCCTAGA	CTCACCCTTGCAGCACTTG	80bp	Cleavage assay, ChIP	Fig.1e, Supp Fig. 3c-e
		ACCATGAACGTGTTCCGAAT	GAGCTCCGCAAAGTTTCAAG	200bp	Resection	Fig. 2e, Supp Fig.4b, 4e
		GGGTATGGAGCTGCCTCTAA	GACAAAGATGGCTGGAGGAG	756bp	ChIP	Supp Fig.3a, 3e
		ACAGATCCAGAGCCACGAAA	CCCACTCTCAGCCTTCTCAG	950bp	Resection	Fig. 2e, Supp Fig.4b
**DSB-2**	chr9:130693171 130693177	TCAAGTCTCAGGGACAAGCC	CTCCCGGGACGATTTCTCG	-2392bp	EdU-IP	Fig. 4b-c, Supp Fig. 5b-c, e,
		GTGGGGGTCCCTTTTCAACC	AAGGCAGAGAGGGAGGAACA	-1321bp	EdU-IP	Fig. 4b-c, Supp Fig. 5b-c, e,
		GCAGTCAGCACCCGAATAGAG	CATACCTACCACGAAGAAGCTGTT	-637bp	EdU-IP	Fig. 4b-c, Supp Fig. 5b-c, e,
		ATGCTTTTCATAGCCGCTGAC	GGCGGATATCCCTCAACACTTC	-341bp	EdU-IP	Fig. 4b-c, Supp Fig. 5b-c, e,
		TCCTCTCTCGGGTCCGC	GGAATGCGGCCAAAGCC	-100bp	EdU-IP	Fig. 4b-c, Supp Fig. 5b-c, e,
		AGACCTCGGTCCGGCT	GCAATGGGGATTTCACGCC	-35bp	EdU-IP	Supp Fig. 5a-b
		CCTACACTTAACCACTGAGCCG	GAGTCTGGCAAGGTGACGAAA	479bp	EdU-IP	Fig. 4b-c, Supp Fig. 5b-c, e,
		CTGTGCCGGATGAGTGTCTATG	ACACACCCATACTCACAGTACCT	841bp	EdU-IP	Fig. 4b-c, Supp Fig. 5b-c, e,
		TATGGGACCAAGCGAGTAGG	GCCTCACACACACACCCATA	863bp	ChIP	Supp Fig. 3a
		GAGGAAGCCATCTGTGACTTAGG	GGACCCTGAGCTTTATCAGTTCTC	2184bp	EdU-IP	Fig. 4b-c, Supp Fig. 5b-c, e,
		GGGTGACAAGAGGGTGACTG	CCGTGGGGTTTTCCTGTTCA	5388bp	EdU-IP	Fig. 4b-c, Supp Fig. 5b-c, e,
**DSB-3**	chr20:30946313 30946319	CCTAGCTGAGGTCGGTGCTA	GAAGAGTGAGGAGGGGGAGT	184bp	ChIP	Supp Fig. 2b, 3a
**DSB-4**	chr17:57184297 57184303	ATCTCTTCAGGTCCAGCGCC	CCACCGTTCGCCCTACTTCT	240bp	EdU-IP	Supp Fig. 5a
**ORI**	chr1:49240607-49244188	TCAGACCCGAGCAATCACAG	CACCCCTATCCCGCATTCAC		EdU-IP	Fig. 4b-c, Supp Fig. 5a-e, Supp
**Actin (ACTB)**	chr7:5566779-5569294	AGCCGGGCTCTTGCCAAT	AGTTAGCGCCCAAAGGACCA		ChIP	Fig.1e
**P0 (RPLP0)**	chr12:12063450 3-120639014	GGCGACCTGGAAGTCCAACT	CCATCAGCACCACAGCCTTC		RT-qPCR	Supp Fig. 3a, 4e, 7a
**BLM**	chr15:91260579 91358686	CCAGTGGTTCCAAGGCAAAG	GCTCCTGATGTCGCTTGAGAA		RT-qPCR	Supp Fig. 3a
**CtIP (RBBP8)**	chr18:20513295 20606449	ACCCCCATGTCCGATACATA	TAGCTGCTTGTTCCATGTGC		RT-qPCR	Supp Fig. 4e
**POLD3**	chr11:7430357 5-74354105	ACCAACAAGGAAACGAAAACAGA	GG TTCCGTGACAGACACTGTA		RT-qPCR	Supp Fig. 7a
**PIF1**	chr15:65107833 65113480	GGGCGAGATGGGATTGTG	GCCCCGAGACACCGATAAGT		RT-qPCR	Supp Fig. 7a
**POLD1**	chr19:50,887,58 0-50,921,275	ATCCAGAACTTCGACCTTCCG	ACGGCATTGAGCGTGTAGG		RT-qPCR	Supp Fig. 7a

Suplementary Table 2: Primer sequences used in this study
