# Supplementary material for: A POLD3/BLM dependent pathway handles DSBs in transcribed chromatin upon excessive RNA:DNA hybrid accumulation
Source: Nat Commun. 2022 Apr 19;13:2012. doi: 10.1038/s41467-022-29629-2 (PMC9019021; doi:10.1038/s41467-022-29629-2)
Supplement: Supplementary file 3 — Reporting Summary [file 41467_2022_29629_MOESM3_ESM.pdf]

nature portfolio

Corresponding author(s): Gaëlle Legube

Last updated by author(s): Mar 18, 2022

Reporting Summary

Nature Portfolio wishes to improve the reproducibility of the work that we publish. This form provides structure for consistency and transparency in reporting. For further information on Nature Portfolio policies, see our Editorial Policies and the Editorial Policy Checklist.

Statistics

For all statistical analyses, confirm that the following items are present in the figure legend, table legend, main text, or Methods section.

n/a	Confirmed	
☐	☒	The exact sample size (*n*) for each experimental group/condition, given as a discrete number and unit of measurement
☐	☒	A statement on whether measurements were taken from distinct samples or whether the same sample was measured repeatedly
☐	☒	The statistical test(s) used AND whether they are one- or two-sided<br>*Only common tests should be described solely by name; describe more complex techniques in the Methods section.*
☒	☐	A description of all covariates tested
☒	☐	A description of any assumptions or corrections, such as tests of normality and adjustment for multiple comparisons
☐	☒	A full description of the statistical parameters including central tendency (e.g. means) or other basic estimates (e.g. regression coefficient) AND variation (e.g. standard deviation) or associated estimates of uncertainty (e.g. confidence intervals)
☐	☒	For null hypothesis testing, the test statistic (e.g. *F*, *t*, *r*) with confidence intervals, effect sizes, degrees of freedom and *P* value noted<br>*Give P values as exact values whenever suitable.*
☒	☐	For Bayesian analysis, information on the choice of priors and Markov chain Monte Carlo settings
☒	☐	For hierarchical and complex designs, identification of the appropriate level for tests and full reporting of outcomes
☐	☒	Estimates of effect sizes (e.g. Cohen's *d*, Pearson's *r*), indicating how they were calculated

Our web collection on statistics for biologists contains articles on many of the points above.

Software and code

Policy information about availability of computer code

Data collection	Bio-Rad CFX Manager version 3.1<br>ChemiDoc™ Touch Imaging System.<br>Image Lab Touch version 1.2.0.12<br>MetaMorph version 7.1.0.0
Data analysis	Custom code will be available at Github upon publication (https://github.com/LegubeDNAREPAIR/BLM_paper).<br>tidyverse 1.3.0 (including ggplot2 R package 3.3.3)<br>Integrated Genome Browser version 9.1.6<br>bwa 0.7.12-r1039<br>samtools 1.9<br>R 3.6.3

For manuscripts utilizing custom algorithms or software that are central to the research but not yet described in published literature, software must be made available to editors and reviewers. We strongly encourage code deposition in a community repository (e.g. GitHub). See the Nature Portfolio guidelines for submitting code & software for further information.

Data

Policy information about availability of data

All manuscripts must include a data availability statement. This statement should provide the following information, where applicable:

- Accession codes, unique identifiers, or web links for publicly available datasets
- A description of any restrictions on data availability
- For clinical datasets or third party data, please ensure that the statement adheres to our policy

New high throughput sequencing data have been deposited to Array Express under the following accession numbers:
E-MTAB-11592 :BLM and RAD51 ChIP-seq, EdU-seq, and DRIP-seq sequencing data

Field-specific reporting

Please select the one below that is the best fit for your research. If you are not sure, read the appropriate sections before making your selection.

☒ Life sciences ☐ Behavioural & social sciences ☐ Ecological, evolutionary & environmental sciences

For a reference copy of the document with all sections, see nature.com/documents/nr-reporting-summary-flat.pdf

Life sciences study design

All studies must disclose on these points even when the disclosure is negative.

Sample size	No sample size calculation was performed. Experiments were generally performed at least 3 times based on standards practice in the field. The number of independent experiments are indicated in the legend of each Figure.
Data exclusions	No data were excluded from analysis.
Replication	ChIP-seq, EdU-seq and DRIP-seq: 3 independent experiments were pooled before sequencing<br>Translocation assays (qPCR) : n≥5<br>RT-qPCR after siRNA treatments : n≥3<br>EdU-qPCR: n≥3<br>ChIP: n=3<br>Colony formation assay: n≥3<br>Western blot: n=4<br>Cell cycle analysis: n=3<br>Repair kinetics and repair fidelity at AsiSI sites: n=3<br>Immunofluorescence: n=3<br>Resection assay: n≥4<br>All replication attempts were successful.
Randomization	Randomization is not relevant because we did not use different experimental groups in our study.
Blinding	Blinding was not relevant to our study since we did not have experimental group to compare.

Reporting for specific materials, systems and methods

We require information from authors about some types of materials, experimental systems and methods used in many studies. Here, indicate whether each material, system or method listed is relevant to your study. If you are not sure if a list item applies to your research, read the appropriate section before selecting a response.

Materials & experimental systems

n/a	Involved in the study	
☐	☒	Antibodies
☐	☒	Eukaryotic cell lines
☒	☐	Palaeontology and archaeology
☒	☐	Animals and other organisms
☒	☐	Human research participants
☒	☐	Clinical data
☒	☐	Dual use research of concern

Methods

n/a	Involved in the study	
☐	☒	ChIP-seq
☒	☐	Flow cytometry
☒	☐	MRI-based neuroimaging

Antibodies

Antibodies used	anti-RNA:DNA hybrid S9.6 purified from a hybridoma ATCC Cat #HB8730 (kind gift from P.Pasero, IGH, France)<br>anti-BLM (Abcam, ab2179, lot #GR3288281-14) Dilution for WB: 1:1000<br>anti-RAD51 (Santa Cruz, SC-8349, lot #E0616)<br>anti-γH2AX (Abcam, ab2893, lot #553424)<br>anti-SETX (Novus Biologicals, NB100-57542, lot #A-4)<br>anti-XRCC4 (Abcam, ab145, lot #GR118332-4)<br>anti-Histone H3 (Abcam ab1791, lot #GR3356864-1)<br>anti-PARP1 (Cell Signaling, 9542, lot #12)<br>anti-alpha Tubulin (Merck, T6557, clone GTU-88) Dilution for WB: 1:10,000<br>anti-Cyclin A2 (Abcam, ab16726, 1:3000)
Validation	S9.6 antibody (kind gift from P.Pasero, IGH, France) previously validated in Cohen et al, Nat Comm 2018<br>All other antibodies used in this study were validated for use in human cells by the manufacturer. Additional informations regarding species, application or relevant citations can be found at the following links:<br>anti-BLM (Abcam, ab2179) https://www.abcam.com/blooms-syndrome-protein-blm-antibody-ab2179.html We additionaly validated this antibody in Fig. S3A.<br>anti-RAD51 (Santa Cruz, SC-8349) previously validated by IF (https://www.scbt.com/p/rad51-antibody-h-92)<br>anti-γH2AX (Abcam 2893) previously validated by IF and WB (https://www.abcam.com/gamma-h2ax-phospho-s139-antibody-ab2893.html)<br>anti-SETX (Novus Biologicals, NB100-57542) previously validated in Cohen et al, Nat Comm 2018 by WB<br>anti-XRCC4 (Abcam, ab145) previously validated by IF and WB (https://www.abcam.com/xrcc4-antibody-ab97351.html). We validated this antibody for ChIP analysis in Aymard et al, NSMB 2014.<br>anti-Histone H3 (Abcam ab1791) previously validated by IF and WB (https://www.abcam.com/histone-h3-antibody-nuclear-marker-and-chip-grade-ab1791.html)<br>anti-PARP1 (Cell Signaling 9542) previously validated by WB (https://www.cellsignal.com/products/primary-antibodies/parp-antibody/9542)<br>alpha Tubulin previously validated by IF and WB (https://www.sigmaaldrich.com/FR/fr/product/sigma/t6557)<br>anti-Cyclin A2 (Abcam, ab16726) https://www.abcam.com/cyclin-a2-antibody-6e6-ab16726.html

Eukaryotic cell lines

Policy information about cell lines

Cell line source(s)	Cell lines developped from U2OS cells (ATCC® HTB-96™) in the Gaelle Legube's laboratory (DIvA cell line and AID-DIvA cell line)
Authentication	Authentication of the U20S cell line was performed by the provider ATCC which uses morphology, karyotyping and PCR based approaches to confirm the identitity of human cell lines. DIvA and AID-DIvA cells derived from U20S cells were not further authenticated.
Mycoplasma contamination	All cell lines (DIvA and AID-DIvA) were regularly tested for absence of mycoplasma contamination by using the MycoAlert Mycoplasma (Lonza). All cell lines used in this study were tested negative for Mycoplasma.
Commonly misidentified lines<br>(See ICLAC register)	No commonly misidentified cell lines were used in the study. U2OS are not registered in ICLAC.

ChIP-seq

Data deposition

☒ Confirm that both raw and final processed data have been deposited in a public database such as GEO.

☐ Confirm that you have deposited or provided access to graph files (e.g. BED files) for the called peaks.

Data access links<br>*May remain private before publication.*	https://www.ebi.ac.uk/arrayexpress/experiments/E-MTAB-11592 :BLM and RAD51 ChIP-seq, EdU-seq, and DRIP-seq data<br>No peak calling was done in this study.
Files in database submission	BLM_normalized_01_02_2018.bw<br>BLM_OHT.clean.fq.gz<br>HJKVVBGX5_RAD51_24H_17s006149-1-1_Clouaire_lane117s006149_sequence.txt.gz<br>HJKVVBGX5_RAD51_24H_17s006149-1-1_Clouaire_lane117s006149_sequence_normalized.bw<br>HKK2NBGX7_BLM_24H_18s002882-1-1_Clouaire_lane118s002882_sequence.txt.gz<br>HKK2NBGX7_BLM_24H_18s002882-1-1_Clouaire_lane118s002882_sequence_normalized.bw<br>HKKWHBGX7_DRIP_C1_24H_18s002884-1-1_Clouaire_lane118s002884_sequence.txt.gz<br>HKKWHBGX7_DRIP_C1_24H_18s002884-1-1_Clouaire_lane118s002884_sequence_normalized.bw<br>HKKWHBGX7_DRIP_STX_24H_18s002886-1-1_Clouaire_lane118s002886_sequence.txt.gz<br>HKKWHBGX7_DRIP_STX_24H_18s002886-1-1_Clouaire_lane118s002886_sequence_normalized.bw<br>HLFCGBGX7_DRIP_BLM_24H_18s002888-1-1_Clouaire_lane118s002888_sequence.txt.gz<br>HLFCGBGX7_DRIP_BLM_24H_18s002888-1-1_Clouaire_lane118s002888_sequence_normalized.bw<br>HLFCGBGX7_DRIP_BLMSTX_24H_18s002890-1-1_Clouaire_lane118s002890_sequence.txt.gz<br>HLFCGBGX7_DRIP_BLMSTX_24H_18s002890-1-1_Clouaire_lane118s002890_sequence_normalized.bw

HVHVTBGXG_EdU-IP_47_20s003953-1-1_Clouaire_lane1EdU47AudemimsiBLM20hOHT_sequence.txt.gz
HVHVTBGXG_EdU-IP_47_20s003953-1-1_Clouaire_lane1EdU47AudemimsiBLM20hOHT_sequence_normalized.bw
HVHVTBGXG_EdU-IP_47_20s003953-1-1_Clouaire_lane1EdU47AudemimsiC20hOHT_sequence.txt.gz
HVHVTBGXG_EdU-IP_47_20s003953-1-1_Clouaire_lane1EdU47AudemimsiC20hOHT_sequence_normalized.bw
HVHVTBGXG_EdU-IP_47_20s003953-1-1_Clouaire_lane1EdU47AudemimsiSETX20hOHT_sequence.txt.gz
HVHVTBGXG_EdU-IP_47_20s003953-1-1_Clouaire_lane1EdU47AudemimsiSETX20hOHT_sequence_normalized.bw
HVHVTBGXG_EdU-IP_47_20s003953-1-1_Clouaire_lane1EdU47AudemimsiSETXBLM20hOHT_sequence.txt.gz
HVHVTBGXG_EdU-IP_47_20s003953-1-1_Clouaire_lane1EdU47AudemimsiSETXBLM20hOHT_sequence_normalized.bw

Genome browser session (e.g. UCSC)

No longer applicable

Methodology

Replicates

3 independent biological replicates were pooled per ChIP-seq analysis

Sequencing depth

Total number of reads: >100 millions
Uniquely mapped reads: >30 millions
Length of reads: 85 bp
Single-end

Antibodies

S9.6 antibody (kind gift from P.Pasero, IGH, France)
BLM (Abcam, ab2179)
RAD51 (Santa Cruz, SC-8349)

Peak calling parameters

No peak calling was done in this study.

Data quality

Read data quality was analyzed with FastQC.

Software

ChIP-seq data were aligned to the reference human genome (hg19) using bwa (http://bio-bwa.sourceforge.net/) and further processed using samtools (http://www.htslib.org/) for duplicate removal (rmdup), sorting (sort) and indexing (index). Coverage for each aligned ChIP-seq dataset (.bam) were computed with the rtracklayer R package and normalized using total read count for each sample.
